# *VdCHS2* Overexpression Enhances Anthocyanin Biosynthesis, Modulates the Composition Ratio, and Increases Antioxidant Activity in *Vitis davidii* Cells

**DOI:** 10.3390/antiox13121472

**Published:** 2024-11-29

**Authors:** Liyuan He, Gongti Lai, Junxuan Lin, Aolin Guo, Fangxue Yang, Ruo Pan, Jianmei Che, Chengchun Lai

**Affiliations:** 1 Institute of Food Science and Technology, Fujian Academy of Agricultural Sciences, Fuzhou 350003, China; 18396531571@163.com (L.H.); laigongti@faas.cn (G.L.); 17857696976@163.com (J.L.); guoaolin3425125034@163.com (A.G.); 13648356144@163.com (F.Y.); rrpanpan@163.com (R.P.); 2 Key Laboratory of Processing of Subtropical Characteristic Fruits, Vegetables and Edible Fungi, Ministry of Agriculture and Rural Affairs of China, Fuzhou 350003, China; 3 Institute of Resources, Environment and Soil Fertilizer, Fujian Academy of Agricultural Sciences, Fuzhou 350003, China

**Keywords:** spine grape (*Vitis davidii* Foëx.), *CHS* gene, light spectrum, anthocyanin biosynthesis, antioxidant

## Abstract

Anthocyanins are significant secondary metabolites that are essential for plant growth and development, possessing properties such as antioxidant, anti-inflammatory, and anti-cancer activities and cardiovascular protection. They offer significant potential for applications in food, medicine, and cosmetics. However, since anthocyanins are mainly obtained through plant extraction and chemical synthesis, they encounter various challenges, including resource depletion, ecological harm, environmental pollution, and the risk of toxic residuals. To address these issues, this study proposes a plant cell factory approach as a novel alternative solution for anthocyanin acquisition. In this study, the *VdCHS2* gene was successfully transformed into spine grape cells, obtaining a high-yield anthocyanin cell line designated as OE1. Investigations of the light spectrum demonstrated that white light promoted spine grape cell growth, while short-wavelength blue light significantly boosted anthocyanin production. Targeted metabolomics analysis revealed that the total anthocyanin content in the OE1 cell line reached 11 mg/g, representing a 60% increase compared to the WT. A total of 54 differentially accumulated metabolites were identified, among which 44 were upregulated. Overexpression of the *CHS* gene enhanced the expression of downstream genes involved in anthocyanin biosynthesis, resulting in the differential expression of *CHI*, *F3Hb*, *F3′5′H*, *DFR4,* and *LDOX*. This led to the differential accumulation of anthocyanin monomers, predominantly consisting of 3-O-glucosides and 3-O-galactosides, thereby causing alterations in anthocyanin levels and composition. Furthermore, the OE1 cell line increased the activity of various antioxidant enzymes, improved the clearance of reactive oxygen species, and reduced the levels of hydrogen peroxide (H_2_O_2_) and malondialdehyde (MDA). The subsequent cultivation of the transformed OE1 cell line, in conjunction with cell suspension culture, established a plant cell factory for anthocyanin production, significantly increasing anthocyanin yield while shortening the culture duration. This study elucidates the molecular mechanisms through which the *VdCHS2* gene influenced anthocyanin accumulation and compositional variations. Additionally, it established a model for a small-scale anthocyanin plant cell factory, thereby providing a theoretical and practical foundation for the targeted synthesis of anthocyanin components and the development and utilization of plant natural products.

## 1. Introduction

Anthocyanins are secondary metabolites classified within the flavonoid family, playing a pivotal role in plant growth and development, as well as in response to environmental stressors, including both biotic and abiotic challenges [1]. Furthermore, anthocyanins confer beneficial effects on human health, evidenced by their roles in antioxidant activity, anti-inflammatory responses, cancer prevention, and cardiovascular protection [2,3]. Chalcone synthase (CHS), a member of the plant-specific type III polyketide synthase superfamily, serves as the primary rate-limiting enzyme in the biosynthetic pathway of flavonoids [4]. It catalyzes the condensation of one molecule of 4-coumaryl CoA with three molecules of malonyl CoA to yield naringin chalcone which acts as a precursor for a diverse array of flavonoid derivatives. CHS is essential for the synthesis of flavanones, flavonoids, quercetin, anthocyanins, and other secondary metabolites [5]. The *CHS* gene was first isolated from ultraviolet-treated suspended culture cells derived from parsley in 1983 [6]. Since then, it has been cloned from various plants including bryophytes [7], algae, gymnosperms [8], and angiosperms [9]. The function of CHS is associated with pigment biosynthesis, defense against pathogenic fungal infections, response to ultraviolet radiation, and tolerance to salt stress [10,11]. Research has demonstrated that the *CHS* gene significantly contributes to anthocyanin accumulation and pigmentation. For instance, the deletion of the *GhCHS* gene resulted in paler coloration compared to wild-type colored cotton [12]. Moreover, overexpression of the *CHS* gene can substantially enhance flavonoid accumulation within plants. For example, introducing *CtCHS* into *Arabidopsis thaliana* led to darker flower phenotypes and a significant increase in flavonoid content [13]. Additionally, overexpression of *EaCHS1* has been shown to considerably improve salt tolerance in crofton weed [14].

Spine grape (*Vitis davidii* Foëx.) is a wild resource within the East Asian grape population and represents a significant germplasm resource in China [15,16]. The majority of its fruits exhibit a purplish-black hue and are rich in anthocyanins, proanthocyanidins, resveratrol, and other bioactive compounds that possess activities such as thrombosis prevention, antioxidant properties, antibacterial effects, and disease resistance [17]. Consequently, spine grape holds considerable promise for applications in food processing, winemaking, and product extraction [18]. Currently, most anthocyanins and other natural pigments are primarily extracted directly from plant tissues. This approach presents challenges, including high costs, resource wastage, and environmental degradation, which significantly restrict their development and application. Additionally, these factors exhibit regional and seasonal variability [19]. Given the aforementioned issues, there is an urgent necessity for innovative alternative solutions to acquire natural products. Consequently, we propose that the extraction of natural secondary metabolites through plant cell culture offers significant feasibility and potential for anthocyanin production.

In this study, a plant expression vector was constructed by utilizing our previously cloned *VdCHS2* gene. A transgenic callus cell line overexpressing *VdCHS2* was induced through *Agrobacterium*-mediated genetic transformation and subsequently treated with various light spectra, including dark, white, blue, and red lights. We investigated the effects of these different light conditions on cell proliferation and the accumulation of anthocyanins, flavonoids, and proanthocyanidins in the *VdCHS2*-transformed cell lines. Metabolite changes in the cell lines were quantitatively analyzed using UPLC-MS/MS. A metabolic model for anthocyanin production in the *VdCHS2*-overexpressing cell lines was established, followed by an analysis of its regulatory mechanisms based on gene expression. Furthermore, we assessed the antioxidant capacity of the *VdCHS2*-transformed cell line and established a small-scale plant cell factory using suspension culture techniques. This study aims to explore the functions and molecular mechanisms of *VdCHS2* gene overexpression and light spectrum treatment in anthocyanin biosynthesis, thereby establishing a foundation for subsequent large-scale production.

## 2. Materials and Methods

### 2.1. Plant Materials and Cell Growth

The spine grape cell line “DLRLH”, derived from the “DLR” cell line induced from immature embryos, was utilized in this study and preserved in our laboratory [20,21]. The cell line was cultured under four distinct light spectrum treatments—darkness, white (mix light), blue (456 nm), and red (637 nm) light—with different technical parameters for each light spectrum, as previously described [22]. The light sources were supplied by different light-emitting diode (LED) lamps (Leesa LED lamp, Zhongshan, China). The culture medium consisted of Murashige and Skoog (MS) solid medium supplemented with 1.0 mg/L 2,4-dichlorophenoxyacetic acid (2,4-D). The cultivation temperature was maintained at 25 ± 2 °C, with relative humidity kept at 50%–60% and a photoperiod cycle of 12 h of light, followed by 12 h of dark treatment. Culture samples were collected on 15, 25, and 35 days, respectively. Each experiment was replicated three times under identical light conditions, with five randomly selected culture bottles pooled during each replication.

### 2.2. Construction of Plant Vector for Overexpression of VdCHS2 Gene

To construct the *VdCHS2* (GenBank accession number OL906400) [23] expression vector, specific primers (Table S1) were employed to amplify the coding sequence of the *VdCHS2* gene, incorporating homologous arms for vector integration. Following the protocol outlined in the pEASY^®^-Basic Seamless Cloning and Assembly Kit (TransGen Biotech, Beijing, China), the target gene fragment was inserted into the pBI121 expression vector at the restriction enzyme sites *Bam*H I and *Xma* I. The connection product between the target gene and the vector was transformed into *Escherichia coli* and identified through colony PCR. The recombinant vector pBI121-*VdCHS2*-GFP was subsequently obtained and verified via sequencing.

### 2.3. Agrobacterium-Mediated Genetic Transformation of Spine Grape Cells

The recombinant vector pBI121-*VdCHS2*-GFP was introduced into *Agrobacterium* GV3101 cells, which were cultured at 28 °C for 48 h and subsequently identified as positive clones via colony PCR. A single colony of *Agrobacterium* was transferred into LB liquid medium containing kanamycin (Kan) and rifampicin (Rif) for further growth. The resulting *Agrobacterium* suspension was then centrifuged at 4000 rpm for 10 min, and the bacterial pellet was resuspended in MS liquid medium supplemented with 20 μmol/L acetosyringone (AS), adjusting the optical density to OD_600_ = 0.6, with a path length of 10.29 mm. The grape cells were infected with the resuspension for 8 min before being placed on sterilized filter paper to remove the excess liquid for 30 s. Subsequently, the cells were transferred to MS solid medium containing 100 μmol/L AS and incubated in darkness for 60–72 h. Following this incubation, the cells were moved to a selection medium (MS + 0.7% agar + 3% sucrose + 1.0 mg/L 2,4-D + 50 mg/L Kan + 100 mg/L timentin) to obtain positive transgenic cell lines.

### 2.4. Screening and Identification of Transgenic Positive Cells

The resistant callus cell lines were screened over three generations, each lasting 20 d. The green fluorescence signal in the resistant cell lines of the callus was detected using a GFP imaging filter with the LUYOR-3415 fluorescent protein excitation light source (LUYOR, Shanghai, China). Following this, genomic DNA was extracted utilizing the CTAB method. Specific primers pBI121-F/R (Table S1) were employed to identify positive cell samples via PCR.

### 2.5. Subcellular Localization Analysis

The identified transgenic positive cell samples were prepared on slides and examined under a confocal laser microscope (Olympus FV1200, Tokyo, Japan) to observe the fluorescence signal and subcellular localization characteristics.

### 2.6. Determination of Anthocyanin, Flavonoid, and Proanthocyanidin Contents

Wild-type (WT) and *VdCHS2*-transformed cell lines cultured under different light conditions for 15, 25, and 35 d were collected. The contents of anthocyanins, flavonoids, and proanthocyanidins were extracted and quantified according to the methods reported in our previous studies [23].

### 2.7. Metabolomic Analysis of Anthocyanins in Spine Grape Cells

Fresh samples of WT and OE1 cell lines cultured under blue light for 35 d were freeze-dried and ground into a powder using a ball mill. A total of 0.5 g of the dry powder was dissolved in 500 μL of extraction solution (50% methanol in water containing 0.1% hydrochloric acid), vortexed for 5 min, sonicated for an additional 5 min, and then centrifuged at 12,000 rpm for 3 min. The supernatant was carefully aspirated and retained, with the extraction process repeated once more. After combining the two supernatants, they were filtered through a 0.22 μm filter membrane before being analyzed using an ultra-high-performance liquid chromatography–tandem mass spectrometry (UPLC-MS/MS) system. Chromatographic determination was conducted by MetWare (Wuhan, China), consisting of ACQUITY BEH C18 (1.7 μm, 2.1 mm × 100 mm). The mobile phases included ultrapure water with 0.5% acetic acid in phase A and methanol with 0.5% acetic acid in phase B; the column temperature was kept at 40 °C, and the sample injection volume was set at 2 μL.

Analyses were conducted using a QTRAP^®^ 6500+ LC-MS/MS system that integrates linear ion trap (LIT) and triple quadrupole (QQQ) scanning techniques. The system was equipped with an ESI Turbo ion spray interface and operated in positive ion mode. Instrument control was facilitated by Analyst 1.6.3 software (Sciex) [24]. Mass spectrometry conditions included an electrospray ion source (ESI) temperature set at 550 °C, a mass spectrometry voltage of 5500 V in positive ion mode, and a curtain gas (CUR) pressure of 35 psi. Scanning detections were performed on the Q-Trap 6500+ utilizing declustering voltages (DPs) and collision energies (CEs). Anthocyanin metabolites were identified through predetermined multiple reaction monitoring (MRM) analysis. Data acquisition was executed via Analyst 1.6.3 software, while Multiquant 3.0.3 software (Sciex) was used for quantitative analysis of metabolites.

### 2.8. RT-qPCR Analysis of Genes Associated with Anthocyanin Synthesis

Total RNA was extracted from the callus treated with various light spectra, and complementary DNA (cDNA) was synthesized using the PrimeScript^TM^ RT Reagent Kit with gDNA Eraser (Takara Bio Inc., Dalian, China). The synthesis of anthocyanins and the expression levels of their structural genes were quantified through real-time fluorescence quantitative PCR. The primers were designed using DNAMAN 8.0 software, with the primer information provided in Table S1, and *alpha-tubulin* (*α-tubulin*) genes were used as internal reference genes. The RT-qPCR was performed using the SYBR Premix Ex Tap II (with TilRNase plus) kit and analyzed on a LightCycler 480 II system (Roche Applied Science, Basel, Switzerland). The reaction conditions included initial denaturation at 95 °C for 10 s, followed by 40 cycles of denaturation at 95 °C for 5 s and annealing/extension at 60 °C for 20 s. The 2^−ΔΔCT^ method was employed to analyze the relative expression levels.

### 2.9. Antioxidant Activity Analysis

Fresh samples from the WT and *CHS2*-overexpressing cell lines (OE1) cultured under blue light for 35 days were collected for the determination of hydrogen peroxide (H_2_O_2_), malondialdehyde (MDA), total antioxidant capacity (T-AOC), reduced glutathione (GSH), and superoxide dismutase (SOD), peroxidase (POD), and catalase (CAT) activities. The levels of H_2_O_2_ and MDA were determined using a hydrogen peroxide assay kit and a malondialdehyde assay kit (Njjcbio, Nanjing, China). The contents of T-AOC and GSH, along with the activities of SOD, POD, and CAT, were analyzed according to the protocols provided by the Total Antioxidant Capacity Assay Kit, Reduced Glutathione Content Assay Kit, Superoxide Dismutase Activity Assay Kit, Peroxidase Activity Assay Kit, and Catalase Activity Assay Kit (Solarbio, Beijing, China), respectively.

### 2.10. VdCHS2 Suspension Cell Culture and Metabolite Determination

The callus grown for up to 20 days was selected and transferred into 30 mL of MS liquid medium (pH 5.8) supplemented with 1.0 mg/L of 2,4-D. The callus was incubated at 25 ± 2 °C and shaken at 120 rpm under a photoperiod of 12 h light and 12 h dark. After an incubation period of 10 days, 10 mL of the cell suspension was transferred to 50 mL of liquid medium to facilitate culture expansion. Samples were collected at 8, 10, and 12 d, and anthocyanin, flavonoid, and proanthocyanidin contents were quantified using the methods described by Lai et al. [25].

### 2.11. Statistical Analysis

All data were analyzed using SPSS statistical 19.0 software to assess significant differences and are expressed as the mean ± standard deviation (SD) (n = 3). One-way ANOVA followed by Duncan’s test was employed for data analysis, with statistically significant differences identified. Statistical analyses and graphical representations were conducted using GraphPad Prism 8 software. Principal component analysis (PCA) and orthogonal partial least squares regression–discriminant analysis (OPLS-DA) were performed via the online data analysis platform Metware Cloud (https://cloud.metware.cn), and correlation heatmaps were generated both within and between groups as network diagrams. These visualizations and further analyses were performed using OmicShare Tools (https://www.omicshare.com/tools).

## 3. Results

### 3.1. Construction of VdCHS2 Overexpression Vector and Genetic Transformation

**Figure 1:**
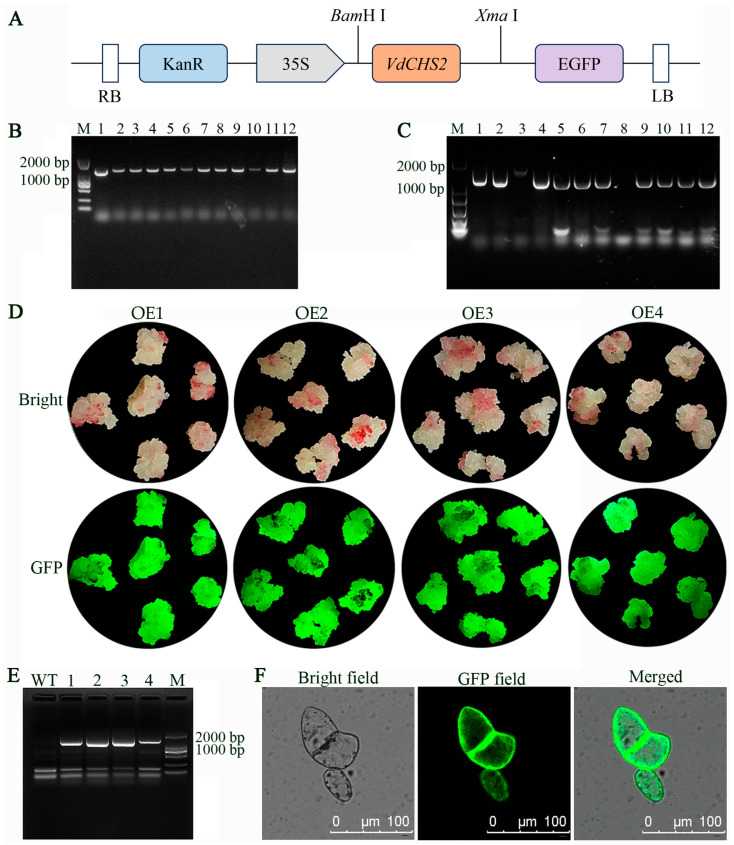
The construction and genetic transformation of the pBI121-*VdCHS2*-GFP vector. (**A**) A schematic diagram of overexpression vector construction. (**B**) PCR analysis of *Escherichia coli*. M: 2000; 1~12: PCR products of pBI121-*VdCHS2* positive colonies. (**C**) PCR analysis of *Agrobacterium* positive colonies. 1~12: PCR products of pBI121-*VdCHS2*-GFP positive colonies. (**D**) Phenotypic characterization of transgenic cell lines. (**E**) PCR assessment of transgenic cell lines. 1~4: transgenic cell lines OE1-4. (**F**) Subcellular localization of VdCHS2 in spine grape cells.

A schematic diagram of the pBI121-*VdCHS2*-GFP vector is presented in Figure 1A. Colony PCR identification revealed that the target size of 12 individual colonies was approximately 1300 bp (Figure 1B), confirming successful ligation and transformation in *Escherichia coli*. The recombinant plasmid was subsequently transformed into *Agrobacterium* GV3101 (Figure 1C). Transgenic cells were examined using a portable fluorescent protein excitation light source. All four transgenic cell lines exhibited distinct green fluorescence signals (Figure 1D). These cell lines, which displayed stable green fluorescence, were subsequently amplified through PCR using specific primers, confirming successful transformation in all four transgenic cell lines (Figure 1E). Additionally, *CHS2* subcellular localization was observed via confocal laser microscopy in transformed spine grape cells, revealing that the VdCHS2 protein was localized in the cytoplasm (Figure 1F).

### 3.2. Light Spectrum Affects Growth and Proliferation of VdCHS2-Transformed Cells

**Figure 2:**
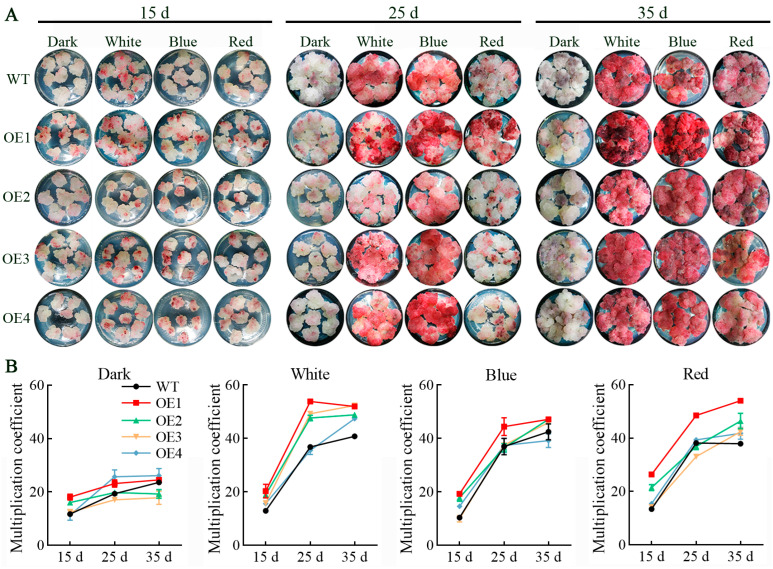
Effect of light spectrum on growth and proliferation of *VdCHS2*-transformed cell lines. (**A**) Phenotypic observations of *VdCHS2*-transformed cell lines under different light spectrum treatments; (**B**) proliferation coefficients of *VdCHS2*-transformed cell lines under different light spectrum treatments.

The transformed cell lines exhibited distinct phenotypic characteristics under varying light spectrum treatments, with their surface color gradually deepening over time (Figure 2A). During the early culture stage (15 d), both the wild-type (WT) and the four transformed cell lines demonstrated a rapid growth trend, displaying a light red coloration on the cell surfaces. Light spectrum treatments revealed that dark conditions inhibited color accumulation on the cell surfaces. After 25 d of culture, all light treatments, except for darkness, led to darker coloration of the cell surface. Among these treatments, the transgenic OE1 cell line displayed the most significant pigment accumulation under blue light, followed by white light treatment. Furthermore, pigment accumulation further continued to intensify, enhancing cell surface coloration over a period of 35 d. The proliferation coefficients of different cell lines under various light spectrum treatments dramatically increased at 25 d (Figure 2B and Table S2) and exhibited slight growth by 35 d in general. Notably, the transformed OE1 cell line exhibited significantly higher levels of pigment accumulation compared to the WT under white light, blue light, and red light, peaking at 53.8 under white light. In conclusion, blue light emerged as the optimal light spectrum for promoting pigment accumulation, followed by white light, red light, and darkness. Additionally, white light proved to be the most effective for enhancing cell proliferation, followed by red light, blue light, and finally darkness. The transformed OE1 cell line demonstrated robust pigment accumulation capabilities alongside relatively high proliferation efficiency.

### 3.3. Light Spectrum Significantly Improved Accumulation of Anthocyanins, Flavonoids, and Proanthocyanidins in VdCHS2-Transformed Cell Lines

**Figure 3:**
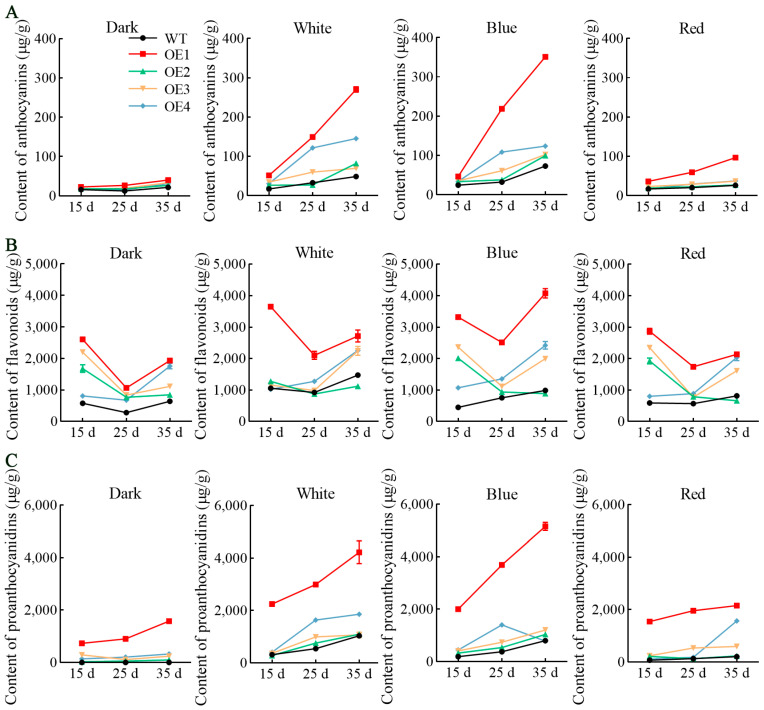
The contents of anthocyanins, flavonoids, and proanthocyanidins in the *VdCHS2*-transformed cell lines under different light spectrum treatments: (**A**) anthocyanins; (**B**) flavonoids; (**C**) proanthocyanidins.

The contents of anthocyanins, flavonoids, and proanthocyanidins in the transformed cell lines were significantly higher than those in the WT under various light spectrum treatments. Notably, the levels of both anthocyanin and proanthocyanidin exhibited a significant upward trend, while the accumulation of flavonoids initially decreased before increasing (Figure 3 and Tables S3–S5). The levels of anthocyanins, flavonoids, and proanthocyanidins in the transformed cell lines were generally higher than those in the WT throughout all three culture stages. The accumulation patterns of anthocyanins and proanthocyanidins showed continuous increases. In contrast, flavonoids displayed a dynamic pattern characterized by an initial decline followed by an upward trend, potentially reflecting their role as intermediate metabolites. Different light spectrum treatments significantly influenced metabolite accumulation, with blue and white light treatments surpassing those of red light and darkness conditions. The metabolites of the transformed OE1 cell line exhibited elevated accumulation levels under blue light treatment, reaching maximum concentrations at 35 d. The results indicated that the levels of anthocyanins, flavonoids, and proanthocyanidins reached 351.1 μg/g, 4069.0 μg/g, and 5155.0 μg/g, respectively, representing increases of 4.5, 4.1, and 6.4-fold compared to those in the WT.

### 3.4. VdCHS2 Affected Anthocyanin Accumulation and Its Component Changes

**Figure 4:**
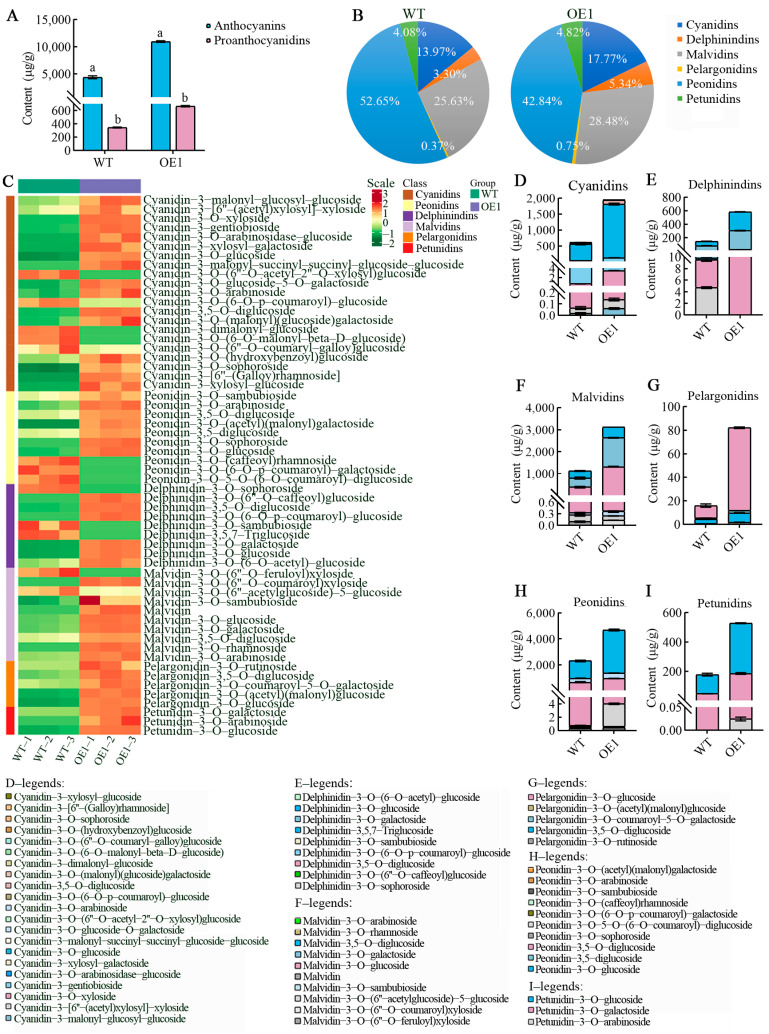
Screening of differential anthocyanin metabolites in spine grape cells (WT and OE1). (**A**) Total content of anthocyanin metabolites in spine grape cells. (**B**) Classification of anthocyanin metabolites. (**C**) Cluster heatmap of anthocyanin metabolites in WT and OE1 cell lines involved in anthocyanin pathway. (**D**–**I**) Total content of each anthocyanin component in spine grape cells (WT and OE1).

Product analysis indicated that *VdCHS2* significantly enhanced the accumulation of metabolites in the transformed OE1 cell line under blue light conditions. To further elucidate the anthocyanin metabolic profile of the high-yield OE1 cell line, both WT and OE1 samples cultured under blue light were analyzed through targeted metabolomics. Data quality assessment revealed significant differences among the samples (Figure S1), with biological replicates showing a high correlation (Figure S2). Additionally, the data exhibited considerable repeatability and reliability (Figure S3). A total of 64 anthocyanin metabolites (Table S6) and their derivatives were identified through metabolomic analysis, which included 58 anthocyanins and 6 proanthocyanidins. The total content of anthocyanins in the OE1 cell line was measured at 10,952.8 μg/g, representing a 60% increase compared to the WT. Additionally, the total content of proanthocyanidins was recorded at 664.5 μg/g, which was 48% higher than that of the WT (Figure 4A). The detected anthocyanin metabolites were primarily categorized into six major classes; specifically, these included 21 cyanidins, 9 delphinidins, 10 malvidins, 5 pelargonidins, and an additional group consisting of 3 peonidins. Notably, peonidins accounted for the highest proportions in both WT and OE1 samples, at approximately 53% and 43%, respectively, while pelargonidins represented the lowest percentage (Figure 4B).

Overexpression of *VdCHS2* significantly increased the accumulation of total anthocyanins in the OE1 cell line, as well as the levels of various components, including cyanidins, delphinidins, malvidins, pelargonidins, peonidins, and petunidins (Figure 4C). Among these components, the accumulation of peonidins reached 4692.8 μg/g, representing a 51% increase, followed by malvidins, while the lowest accumulation was observed for pelargonidins, at only 82.2 μg/g. The observed difference in anthocyanin content between the two cell lines can be attributed to the differential accumulation of anthocyanin monomers. The monomers that exhibited significant differential accumulation in cyanidins included cyanidin-3-O-glucoside, cyanidin-3,5-O-diglucoside, and cyanidin-3-gentiobioside. Among these, the accumulation of cyanidin-3-O-glucoside was the highest, reaching 1684.0 μg/g DW, which represented an increase of 69% (Figure 4D). In the case of delphinidins, significant differential accumulation was observed for delphinidin-3-O-galactoside, delphinidin-3-O-glucoside, and delphinidin-3,5-O-diglucoside, with delphinidin-3-O-galactoside showing the highest accumulation at 280.5 μg/g, an increase of 76% (Figure 4E). Notable differences in the accumulation of malvidin were detected in malvidin-3-O-galactoside, malvidin-3-O-glucoside, and malvidin-3,5-O-diglucoside, with malvidin-3-O-galactoside reaching the highest level of 1313.4 μg/g, an increase of 69% (Figure 4F). Significant differences in pelargonidin monomers were also observed in the accumulation of pelargonidin-3-O-glucoside, pelargonidin-3,5-O-diglucoside, and pelargonidin-3-O-rutinoside, with pelargonidin-3-O-glucoside accumulating to 70.2 μg/g, an increase of 85% (Figure 4G). The most significant differences in peonidin accumulation were found for peonidin-3-O-glucoside, peonidin-3,5-diglucoside, and peonidin-3.5-O-giglucoside, with peonidin-3-O-glucoside attaining the highest level at 3338.7 μg/g DW, an increase of 60% compared to the WT (Figure 4H). The most significant increase in petunidin accumulation was observed for petunidin-3-O-glucoside, which increased by 62% compared to the WT (Figure 4I). The analysis of the anthocyanin composition revealed that the monomer contents of 3-O-glucoside and 3-O-galactoside in the transformed OE1 cell lines were 7032.0 μg/g and 1779.4 μg/g DW, respectively, which increased by 2.9-fold and 3.4-fold compared to the WT (Table S7). In summary, the *VdCHS2* gene significantly enhances anthocyanin production and promotes the differential synthesis and stable accumulation of anthocyanin components, primarily through the modification of 3-O-glucoside and 3-O-galactoside.

### 3.5. Overexpression of VdCHS2 Affects Expression of Anthocyanin Synthesis Genes and Metabolites

**Figure 5:**
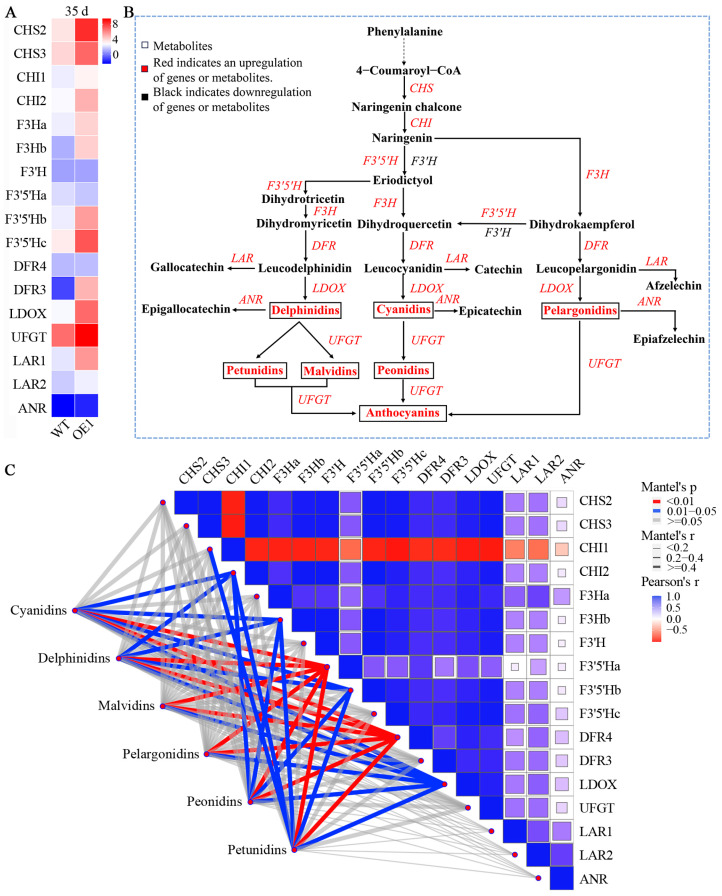
Gene expression and network correlation analysis involved in anthocyanin biosynthesis. (**A**) Gene expression levels after culturing for 35 d. (**B**) Integrative analysis of genes expression and anthocyanin accumulation. (**C**) The correlation heatmap illustrating the relationship between anthocyanin metabolites and 17 gene modules. Mantel’s P indicates the significance level of the correlations between genes and metabolites, while Mantel’s r reflects the strength of these correlations. The thickness of the connecting lines corresponds to the overall correlation coefficient, whereas the color of these lines denotes the results of significance testing for the correlations between variables. Pearson’s correlation coefficient evaluates the significance level of relationships among genes. The coloration of cell blocks represents correlation coefficients, with blue indicating positive correlations and red signifying negative correlations.

To elucidate the molecular mechanisms underlying anthocyanin biosynthesis and accumulation induced by the *VdCHS2* gene, the gene expression involved in anthocyanin biosynthetic pathways was investigated. The results demonstrated that overexpression of *CHS2* enhanced the expression of downstream genes (Figure 5); 15 genes were significantly upregulated in the OE1 cell line, including *CHS*, *CHI*, *F3H*, *DFR*, *LDOX*, *LAR,* and *UFGT*. Notably, only the *F3’H* gene was significantly downregulated (Figure 5A). The expression levels of *CHS2* and *UFGT* were markedly increased, with increases of 27.2- and 60.5-fold, respectively. The high expression of *CHS2* significantly enhanced the expression of downstream key genes, particularly enhancing the expression of *UFGT*, which facilitated the accumulation of anthocyanins in a more stable form via glycoside modification. The gene expression results were consistent with the accumulation patterns of anthocyanin components and monomer profiles (Figure 5B). Therefore, overexpression of *VdCHS2* markedly enhanced the expression of critical downstream genes involved in anthocyanin synthesis and modification, thereby promoting both the synthesis and differential accumulation of components.

The results of quantitative PCR demonstrated that *VdCHS2* promoted the expression of downstream genes. To further analyze the interaction between gene expression and anthocyanin components, network correlation analysis revealed that all six anthocyanin components exhibited a significant correlation with *F3′5′Ha*, *DFR4*, and *LDOX*. Cyanidins, delphinidins, peonidins, and petunidins showed significant correlations with *CHI2*, *F3Hb,* and *F3′5′Hb*, while petunidins were significantly related to *CHI1*. Among these genes, *CHI1*, *CHI2*, *F3Hb*, *F3′5′Hb,* and *LDOX* presented a positive correlation with anthocyanin levels, while *F3′5′Ha* and *DFR4* exhibited a negative correlation. However, neither the upstream genes *CHS* and *F3′H* nor the downstream genes *UFGT*, *LAR,* and *ANR* observed any discernible correlations with anthocyanin components. In conclusion, overexpression of *CHS2* affected the differential expression of *CHI*, *F3Hb*, *F3′5′H*, *DFR4,* and *LDOX*, which indirectly led to the differential accumulation of anthocyanin components between the WT and *CHS2*-overexpressing cell lines, with *F3′5′Ha* and *DFR4* being strongly related to the specific accumulation of these components.

### 3.6. Overexpression of VdCHS2 Significantly Increased Antioxidant Activity in Spine Grape Cells

**Figure 6:**
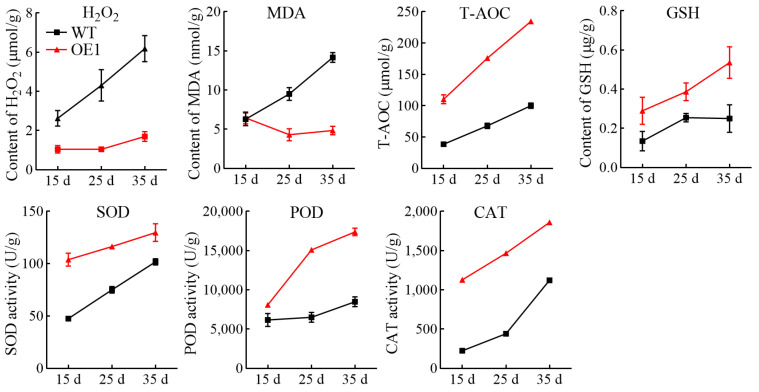
Effects of overexpression of *VdCHS2* on antioxidant capacity and antioxidant oxidase activity in spine grape cells.

To further investigate the radical scavenging ability of *CHS2*-overexpressing cells, the levels of hydrogen peroxide (H_2_O_2_) and malondialdehyde (MDA), total antioxidant capacity (T-AOC), reduced glutathione (GSH), superoxide dismutase (SOD), peroxidase (POD), and catalase (CAT) were assessed. The results indicated that the levels of H_2_O_2_ and MDA in the OE1 cell line were significantly lower than those in the WT (Figure 6). H_2_O_2_ and MDA levels in the WT exhibited a continuous accumulation pattern with prolonged culture duration. The OE1 cell line, on the other hand, did not exhibit any appreciable accumulation and even showed a slight decrease during the initial phases. Specifically, the levels of both H_2_O_2_ and MDA were 0.7-fold those in the WT. This decrease may be attributed to the elevated levels of antioxidant compounds, such as anthocyanins and proanthocyanidins, present in the OE1 cell line, which enhanced the clearance of reactive oxygen species and protected membrane lipids. Additionally, the levels of T-AOC, GSH, SOD, POD, and CAT in both cell lines increased with prolonged culture duration. Notably, the antioxidant capacity and the oxidase activity in the OE1 cell line were significantly greater than those in the WT. Overexpression of *CHS2* resulted in a 2.3-fold increase in the total antioxidant capacity. In conclusion, *CHS2* overexpression promoted the accumulation of anthocyanins and other antioxidant compounds, improved the activity of antioxidant oxidase, and enhanced the antioxidant capacity of spine grape cells, thereby increasing the scavenging of reactive oxygen species and protecting membrane lipids from oxidative damage.

### 3.7. Construction of Anthocyanin Plant Cell Factory Based on CHS2 Overexpression

**Figure 7:**
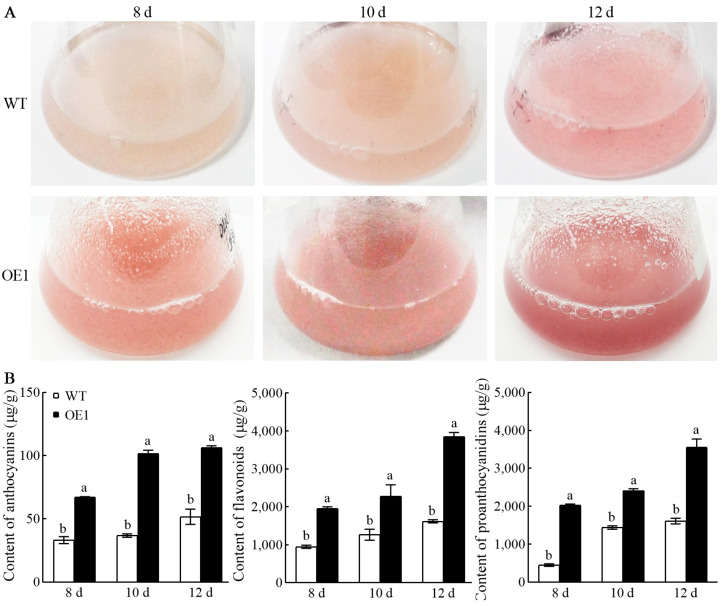
Suspension culture of transgenic OE1 cell line. (**A**) Cultivation of suspended cells for 8, 10, and 12 d under WT and OE1 cell line. (**B**) Contents of anthocyanins, flavonoids, and proanthocyanidins. The different letters indicate significant difference at 0.05 levels (*p* < 0.05).

In the OE1 cell line, *CHS* overexpression improved anthocyanin’s and other antioxidant compounds’ accumulation and antioxidant ability. To efficiently obtain natural anthocyanin products, cell suspension culture was employed (Figure 7). The findings showed that as the culture duration increased, the color of the suspended cells in the WT and transformed OE1 cell line deepened progressively. Specifically, after culturing the OE1 cell line for 8, 10, and 12 d, the color of the suspensions appeared as light red, red, and dark red hues, respectively. Each stage exhibited a significantly deeper coloration compared to the WT, indicating a higher level of anthocyanin accumulation (Figure 7A). Further analysis of antioxidant metabolite contents in cell lines after different culture durations revealed that the anthocyanin, flavonoid, and proanthocyanidin levels in the OE1 cell line were significantly higher than those in the WT (Figure 7B), peaking at 12 d with concentrations of 106.5 μg/g, 3849.4 μg/g, and 3560.9 μg/g, representing 2.0, 2.4, and 2.2-fold increases, respectively. The suspension culture method ensured an adequate supply of nutrients and uniform light for the cells or cell masses, thereby significantly reducing the culture cycle compared to solid culture, which typically requires 25–30 d. Overall, overexpression of *CHS* in conjunction with suspension cell culture markedly increased the content of anthocyanins, flavonoids, and proanthocyanidins while substantially shortening the culture cycle.

## 4. Discussion

### 4.1. The CHS Gene Promotes the Growth and Maturation of Spine Grape Cells

The *CHS* gene is widely distributed in plants and plays a crucial role in plant growth and development. Some studies have indicated that overexpression of the *CHS* gene can not only enhance plant growth but also influence plant coloration [26]. In rice seedlings, the knockout of the *CHS1* gene resulted in a significant increase in grain length [27]. In comparison to the overexpressed *CHS* line, *Arabidopsis* plants with gene knockout exhibited reduced growth and fewer rosette leaves [28]. Overexpression of *CpCHS1* can alleviate drought-induced growth inhibition by increasing the germination rate and fresh weight of tobacco seeds, while transgenic *CHS* plants did not show significant differences in plant height, stem girth, or other morphological characteristics compared to non-transgenic plants [29]. Additionally, studies have shown that transgenic *HVCHS* can significantly enhance the coloration of tobacco plants [30]. In this study, compared to the WT, the surface coloration of the four transformed cell lines exhibited deeper pigment accumulation as the culture duration was extended. Furthermore, the surface cells displayed a more pronounced mature state, with the transformed OE1 cell line demonstrating the most significant color change effect. The proliferation coefficient of the transformed cell lines initially rose significantly before leveling off as the culture duration continued to extend, with the OE1 cell line presenting the highest proliferation coefficient. Therefore, the *CHS* gene contributed to an increase in the fresh weight of the cell lines and accelerated its maturation to a certain extent. This suggests that overexpression of *VdCHS2* could further shorten the cell culture cycle and promote vigorous cell growth.

### 4.2. Short-Wavelength Light Promoted the Synthesis and Accumulation of Secondary Metabolites Such as Anthocyanins

Plant growth regulators, including 5-aminolevulinic acid, abscisic acid, ethylene, and melatonin, along with environmental factors such as light spectrum, temperature, and water stress, play a crucial role in regulating metabolite synthesis. Light is one of the critical ecological factors influencing the type and content of secondary metabolites, including anthocyanins [31]. Light spectrum, intensity, and duration can all affect anthocyanin accumulation, with light spectrum being particularly decisive [32]. Although the impact of different light spectrum treatments on anthocyanin accumulation in plants is widely recognized, the underlying molecular mechanisms remain to be elucidated. Research has demonstrated that blue light treatment significantly increases the anthocyanin content in purple pepper fruit [33], as well as in *Mangifera indica* fruits [34] and *Solanum melongena* [35]. Conversely, red light has been shown to significantly increase anthocyanin content in strawberries, while exposure to blue film reduced it [36]. Additionally, blue light induction has been found to substantially enhance anthocyanin accumulation in *Arabidopsis thaliana* [37]. In the present study, spine grape cells were cultivated under dark, white (mixed), blue (short-wavelength), and red (long-wavelength) light conditions. The results indicated that treatments with different light spectra significantly promoted the accumulation of anthocyanins, flavonoids, and proanthocyanidins, with blue light treatment exhibiting the most pronounced effect, followed by white light. This was consistent with results showing that blue light exerted the strongest promoting effect on anthocyanin synthesis in grapefruits [38]. This suggests that short-wavelength blue light distinctly influences anthocyanin synthesis across various plant species. Furthermore, as the culture period extended, the trend of flavonoids in cell lines treated with different light qualities initially increased before subsequently decreasing. In contrast, both anthocyanins and proanthocyanidins exhibited a significant increase over time. Interestingly, the accumulation of secondary metabolites, particularly anthocyanins, was the highest in the OE1 cell line under blue light treatment. Therefore, short-wavelength light effectively promotes the accumulation of secondary metabolites, including anthocyanins, in spine grape cells. To optimize anthocyanin production, it is advisable to first culture the cells under white light to enhance biomass. Subsequently, in the later stages, short-wavelength light, such as blue light, should be employed to induce anthocyanin synthesis and accumulation.

### 4.3. The CHS Gene Increased Anthocyanin Accumulation and Affected the Composition of Anthocyanins

Anthocyanins are flavonoid compounds widely distributed in plants [39], primarily existing in glycoside forms that combine with glucose, rhamnose, xylose, galactose, and arabinose to form stable products [40]. CHS is the first key rate-limiting enzyme that facilitates the conversion of phenylalanine to chalcone and plays a crucial role in anthocyanin biosynthesis [41]. In this study, overexpression of *VdCHS2* significantly enhanced anthocyanin accumulation in spine grape cells at various culture stages. Research indicated that overexpression of the *RdCHS1* gene markedly elevated the expression of the endogenous tobacco gene *NtCHS*, thereby promoting anthocyanin accumulation [42]. Compared to wild-type hairy roots, overexpression of the *CHS* gene significantly increased the flavonoid content in the hairy roots of *Glycyrrhiza uralensis* [43], and the expression level of the *CHS* gene was positively correlated with the color depth of *Glycine max* [44]. Overexpression of *SoCHS1* in tobacco resulted in darker corolla coloration, suggesting that this gene is involved in anthocyanin synthesis [45], which aligns with the findings of this study. Consequently, the *VdCHS2* gene significantly induces the biosynthesis of secondary metabolites, such as anthocyanins in spine grape cells.

Simultaneously, overexpression of *VdCHS2* significantly influenced the accumulation of anthocyanin components by upregulating other structural genes in the anthocyanin biosynthesis pathway, thereby enhancing anthocyanin accumulation. Studies have shown that cyanidin-3-O-rutinoside is the primary contributor to the purple appearance of *Triticum aestivum* kernels [46]. Furthermore, it has been demonstrated that the main components of purple *Triticum aestivum* kernels include cyanidin-3-glucoside, cyanidin-3-(6-malonylglucoside), cyanidin-3-rutinoside, peonidin-3-glucoside, and peonidin-3-malonylglucoside [47]. The anthocyanin metabolites delphinidins, peonidins, and malvidins are primarily responsible for the purple and dark coloration of *Dioscorea esculenta*, while cyanidins and malvidins contribute predominantly to its red coloration [48]. Research indicated that concentrations of total anthocyanins, pelargonidin-3-gluc, and pelargonidin-3-malonylgluc in strawberries significantly increased following treatments with blue and red lights [49]. In this study, a total of 64 differential anthocyanin metabolites were identified in the OE1 cell line compared to the WT, including 21 cyanidin metabolites, 9 delphinidin metabolites, 10 malvidin metabolites, 5 pelargonidin metabolites, 10 peonidin metabolites, 3 petunidin metabolites, and 6 proanthocyanidin metabolites. Overexpression of *VdCHS2* led to an increase in total anthocyanin accumulation in the OE1 cell line and elevated the levels of various components, including cyanidins, delphinidins, malvidins, pelargonidins, peonidins, and petunidins. The high expression of *CHS2* enhanced the expression levels of downstream key synthetic genes, particularly enhancing the expression of *UFGT*. The high expression of these genes promoted the accumulation of anthocyanins in a more stable form through glucoside modification. The primary modification modes of anthocyanin monomers are 3-O-glucoside and 3-O-galactoside. Correlation analysis revealed that *CHI*, *F3Hb*, *F3′5′H*, *DFR4*, and *LDOX* were significantly correlated with the accumulation of anthocyanin components. Therefore, *VdCHS2* promotes the stable accumulation of anthocyanins through the expression of *CHI*, *F3Hb*, *F3′5′H*, *DFR4*, and *LDOX* genes, while the modification mode dominated by 3-O-glucoside and 3-O-galactoside can further influence the changes in anthocyanin components.

### 4.4. The CHS Gene Increased the Antioxidant Activity of Spine Grape Cells

Plants are often subjected to abiotic stresses during their growth and development, such as low temperature, drought, high salinity, and senescence. When exposed to these stresses, plants typically generate a substantial amount of reactive oxygen species, leading to oxidative stress [50]. Phenylpropanoid metabolism constitutes a crucial secondary metabolic pathway in plant defense mechanisms. It facilitates the synthesis of flavonoids, which provide antiviral and antifungal properties, as well as other phenolic compounds that reinforce cell walls to physically isolate oxidation reactions. CHS is a key enzyme involved in the catalysis of the synthesis of these products [51]. In this study, the overexpressed *VdCHS2* exhibited lower levels of MDA and H_2_O_2_ compared to the WT, as well as enhanced T-AOC and increased activities of GSH, POD, CAT, and SOD. MDA serves as a critical parameter reflecting the potential antioxidant capacity of the organism, with its levels directly indicating the extent of damage to the cell membrane [52]. The significantly reduced MDA content indicated less damage to the cell membrane, which can be attributed to the fact that the ROS clearance system in plants predominantly relies on antioxidant enzymes, including CAT, SOD, and POD [53]. These enzymes are instrumental in eliminating H_2_O_2_, effectively scavenging free radicals, with their activity levels being closely associated with the stress resistance of plants [54]. Studies have demonstrated that the upregulation of *CHS* resulted in a significant enhancement of flavonoids in the hair root of *Isatis indigoticus*, which mitigated excess ROS production [55]. In summary, overexpression of *CHS2* promoted the accumulation of anthocyanins and other antioxidant compounds, enhanced the activity of antioxidant enzymes, and subsequently improved the scavenging capacity for free radicals, thereby providing protective effects on cells.

## 5. Conclusions

In this study, we genetically transformed spine grape cells with the *VdCHS2* gene, resulting in the establishment of a high-yield anthocyanin cell line designated as OE1, which achieved an anthocyanin content of 11 mg/g. Different light spectrum treatments revealed that white light facilitated the growth of spine grape cells, while short-wavelength blue light was more effective in promoting anthocyanin accumulation. Targeted metabolomics analysis indicated that the total anthocyanin content in the OE1 cell line was 60% higher than that in the wild type (WT), with the highest peonidin-3-O-glucoside content reaching 3.3 mg/g. Overexpression of the *VdCHS2* gene enhanced the stable accumulation of anthocyanins through predominant modifications of 3-O-glucoside and 3-O-galactoside, and significant correlations were observed between *F3′5′Ha* and *DFR4* in relation to the differential accumulation of anthocyanin compositions. Additionally, overexpression of *VdCHS2* increased the activity of various antioxidant enzymes in the OE1 cell line, resulting in decreased levels of hydrogen peroxide (H_2_O_2_) and malondialdehyde (MDA), as well as improved scavenging of oxygen radicals and oxidative protection of membrane lipids. The establishment of anthocyanin-producing plant cell factories utilizing high-yield cell lines led to a remarkable increase in anthocyanin production and a reduction in the culture period. This study offers insights into the regulatory mechanisms of the *CHS2* gene in anthocyanin synthesis in plants, providing theoretical guidance for the targeted production of specific metabolite components.

## Data Availability

The data are contained within the article and Supplementary Materials.

## Supplementary Materials

The following supporting information can be downloaded at: https://www.mdpi.com/article/10.3390/antiox13121472/s1, Word file: Table S1: The primers for vector construction and RT-qPCR; Table S2: The multiplication coefficient of transgenic cell lines under light quality treatment; Table S3: Anthocyanins content in transgenic cell lines under light quality treatment (μg/g); Table S4: Flavonoids content in transgenic cell lines under light quality treatment (μg/g); Table S5: Proanthocyanidins content in transgenic cell lines under light quality treatment (μg/g); Table S6: Total anthocyanin metabolites in the WT and OE1 cell lines; Table S7: Glycosylation modification of anthocyanin components; Figure S1: OPLS-DA score map of metabolites PCA of metabolome data; Figure S2: Pearson’s correlation analysis of each sample in the transcriptome data; Figure S3: Superposed graph of total ions current in quality control samples.
