# Supplementary material for: VdCHS2 Overexpression Enhances Anthocyanin Biosynthesis, Modulates the Composition Ratio, and Increases Antioxidant Activity in Vitis davidii Cells
Source: Antioxidants (Basel). 2024 Nov 29;13(12):1472. doi: 10.3390/antiox13121472 (PMC11673275; doi:10.3390/antiox13121472)
Supplement: Supplementary file 1 [file antioxidants-13-01472-s001.zip › antioxidants-3317274-supplementary.pdf]

Supplementary material

VdCHS2 Overexpression Enhances Anthocyanin Biosynthesis, Modulates the Composition Ratio, and Increases Antioxidant Activity in *Vitis davidii* Cells

Table S1 The primers for vector construction and RT-qPCR

Gene	Sequence (5′-3′)	Function
VdCHS2-F	GAACACGGGGGACTCTAGAGATGGTGTC AGTGGGGGAAAT	Vector construction
VdCHS2-R	ACCATGGTGGCGACCGGTACGTGAGTCG ATTGTGTAGCAA	
pBI121-F	GAACACGGGGGACTCTAGAG	
pBI121-R	ACCATGGTGGCGACCGGTAC	
VdCHS2-qPCR-F	GTCCCAGGGTTGATTTCC	Real-time quantitative PCR
VdCHS2-qPCR-R	GCTCTCAGTTTCTCTTCCTTCA	
VdCHS3-qPCR-F	CCCGTGTCCTTGTTGTCTG	
VdCHS3-qPCR-R	GATTGTCTGGGCTGCTGA	
VdCHI1-qPCR-F	GCTTTCTCTTCTCTCCACCTGT	
VdCHI1-qPCR-R	CACCTCATCACCACCATACC	
VdCHI2-qPCR-F	GTTCCTACGCTCGCCGTCAA	
VdCHI2-qPCR-R	ATACTGGCGACCCGTCAAAG	
VdF3Ha-qPCR-F	CGGTTTGGAAGGGAAGGTC	
VdF3Ha-qPCR-R	GCCTGGAACATCATTGGG	
VdF3Hb-qPCR-F	GAAGATTGTGGAGGCGTGT	
VdF3Hb-qPCR-R	TGGAGATGACTGGAGACGA	
VdF3'H-qPCR-F	GATTCAGGAGCGGAGACTC	
VdF3'H-qPCR-R	AAGCCCATACGGAGACTC	
VdF3'5'Ha-qPCR-F	CAAACCTTCGCTCATCTACC	
VdF3'5'Ha-qPCR-R	GCAGCAAACTCAAGTAAGA	
VdF3'5'Hb-qPCR-F	GCGGGATGAAGCATTTACAT	
VdF3'5'Hb-qPCR-R	TGATGAAGTGTCTGTTCCTGC	
VdF3'5'Hc-qPCR-F	CAAGTGATTGGAAGGAGCC	
VdF3'5'Hc-qPCR-R	TGAGACACGAGGAAGGTTC	
VdDFR4-qPCR-F	TGCTGATGAAGGAAGTTTCG	
VdDFR4-qPCR-R	GCGGAGGATGTGAATACAAG	
VdDFR3-qPCR-F	ACCATCACAGCCAAGGACA	
VdDFR3-qPCR-R	CATAAACCCAACCACCCAC	
VdLDOX-qPCR-F	GGAAGTTGGTGGGATGGA	
VdLDOX-qPCR-R	GTGGAGGATGAAGGTGAGAG	

VdUFGT-qPCR-F	GATTGGAGTTTCAGGCATTCAAG
VdUFGT-qPCR-R	CATGCGTGAGAAGAGCGAGT
VdLAR1-qPCR-F	GGTTTCATTGGTCGTCAGTTCGT
VdLAR1-qPCR-R	GCCTCCTGCTCGTTTATCA
VdLAR2-qPCR-F	CAATACCCACCCTGCTGA
VdLAR2-qPCR-R	TTCTCCCACAGTGATGCC
α-*Tubulin*-qPCR-F	GTTCTCGCGCATTGACCATA
α-*Tubulin*-qPCR-R	CAGCCAGATCTTCACGAGCTT

Table S2 The multiplication coefficient of transgenic cell lines under light quality treatment

Culture duration	Sample	Dark	White	Blue	Red
15 d	WT	9.41±0.04	10.18±0.19	10.39±0.21	13.45±0.46
	OE1	20.85±1.08	20.3±1.81	19.26±0.23	26.37±0.29
	OE2	16.02±0.59	18.94±0.34	17.84±0.96	21.44±0.79
	OE3	12.39±0.87	15.8±0.78	9.93±0.87	14.2±0.2
	OE4	11.37±1.37	15.61±0.38	14.6±0.23	15.46±0
25 d	WT	19.34±0.63	36.79±0.64	43.19±1.44	38.19±0.64
	OE1	23.16±0.96	53.82±0.16	50.96±2.67	48.56±0.27
	OE2	28.13±0.19	47.67±0.73	36.6±1.09	36.87±0.84
	OE3	19.63±0.03	49.24±0.33	37.47±0.33	33.11±0.29
	OE4	29.46±1.97	35.57±1.29	42.93±2.16	39.33±0.19
35 d	WT	23.6±0.31	40.79±0.67	30.51±0.83	37.37±0.82
	OE1	24.57±0.23	52.01±0.3	47.16±0.27	54.06±0.63
	OE2	19.31±1.14	48.83±0.14	47.13±0.33	46.39±2.07
	OE3	15.37±0.66	52.31±0.57	46±0.37	32.03±1.57
	OE4	24.07±0.19	47.46±0.03	39.17±2.06	38.16±2.07

Table S3 Anthocyanins content in transgenic cell lines under light quality treatment (μg/g)

Culture duration	Sample	Dark	White	Blue	Red
15 d	WT	21.7±0.55	17.46±0.3	24.32±1.05	26.32±0.58
	OE1	40.54±1.05	51.96±1.3	46.6±1.57	36.24±0.83
	OE2	26.62±0.89	26.86±1.96	33.68±0.59	27.59±0.55
	OE3	33.85±0.61	34.95±0.8	36.02±0.55	29.08±0.62
	OE4	31.42±1.44	30.99±1.1	34.37±0.61	29.48±0.05
25 d	WT	16.02±0.61	33.31±0.3	32.37±0.8	16.97±0.32
	OE1	22.95±1.43	149.59±1.05	219.2±3.35	59.61±0.78
	OE2	18.47±0.03	27.44±0.94	38.02±0.3	19.72±0.53
	OE3	19.42±0.32	59.91±0.62	61.25±1.1	23.31±0.33
	OE4	16.19±0.27	121.94±1.09	108.62±1.61	17.29±0.32
35 d	WT	12.77±0.32	48.67±0.53	73.14±0.8	20.37±0.93
	OE1	26.77±0.53	270.22±5.7	351.05±2.9	97.01±1.52
	OE2	17.96±0.3	81.94±2.14	99.81±0.91	22.9±0.91
	OE3	17.03±0.53	69.63±1.1	102.77±0.78	36.82±0.61
	OE4	15.31±0.61	145.31±1.69	124.45±1.82	37.29±0.32

Table S4 Flavonoids content in transgenic cell lines under light quality treatment (μg/g)

Culture duration	Sample	Dark	White	Blue	Red
15 d	WT	578.82±7.84	1057.25±22.18	450.72±41.17	586.67±25.62
	OE1	2604.97±26.66	3645.49±12.81	3316.08±22.18	2871.63±77.2
	OE2	1674.25±99.48	1276.86±33.89	2008.89±57.75	1925.23±73.95
	OE3	2197.12±26.66	2244.18±114.79	2359.22±22.18	2348.76±39.13
	OE4	806.27±46.18	1018.04±7.84	1067.71±51.76	801.05±26.66
25 d	WT	278.17±14.79	1470.33±57.75	753.99±19.56	570.98±22.18
	OE1	1062.48±32.23	2103.01±107.41	2510.85±26.66	1739.61±23.53
	OE2	764.44±29.58	869.02±33.89	936.99±19.56	785.36±32.23
	OE3	837.65±25.62	1156.6±7.39	1112.16±39.22	774.9±44.37
	OE4	672.94±7.84	1271.63±41.17	1350.07±32.23	884.71±15.69
35 d	WT	638.95±63.18	780.13±39.13	984.05±41.17	816.73±19.56
	OE1	1930.46±53.32	2714.77±154.93	4069.02±122.18	2134.38±63.18
	OE2	842.88±19.56	1120±44.37	884.71±31.37	659.87±26.66
	OE3	1109.54±14.79	984.05±60.53	1993.2±32.23	1611.5±7.39
	OE4	1752.68±70.54	2254.64±26.66	2421.96±101.66	2040.26±81.34

Table S5 Proanthocyanidins content in transgenic cell lines under light quality treatment (μg/g)

Culture duration	Sample	Dark	White	Blue	Red
15 d	WT	0±0	319.41±11.09	192±29.03	72±4.44
	OE1	1584.59±27.48	2254.22±7.26	2005.33±19.2	1546.07±29.33
	OE2	55.7±8.38	286.81±11.09	325.33±14.52	212.74±4.19
	OE3	292.74±43.75	369.78±7.26	411.26±11.09	594.96±35.8
	OE4	138.67±26.17	409.78±13.33	443.85±18.26	116.44±4.44
25 d	WT	0±0	547.56±12.57	378.67±8.89	134.22±13.33
	OE1	903.11±35.56	2997.93±41.27	3694.22±14.52	1960.89±37.71
	OE2	27.56±13.33	760.89±26.17	535.7±27.48	129.78±8.89
	OE3	116.44±13.33	994.96±22.17	743.11±33.26	245.33±8.89
	OE4	205.33±4.44	1643.85±18.26	1400.89±69.23	178.67±4.44
35 d	WT	0±0	1039.41±32.73	800.89±13.33	209.78±17.78
	OE1	729.78±66.67	4224.59±353.28	5154.96±122.81	2153.48±16.76
	OE2	97.19±32.73	1101.63±30.22	1036.44±7.26	236.44±19.2
	OE3	242.37±18.26	1072±83.7	1211.26±60.43	538.67±26.17
	OE4	325.33±17.78	1860.15±4.19	787.56±8.89	1572.74±22.17

Table S6 Total anthocyanin metabolites in the WT and OE1 cell lines

Metabolites	WT (μg/g)	OE1 (μg/g)
Cyanidin-3-malonyl-glucosyl-glucoside	0.02±0	0.06±0.01
Cyanidin-3-[6"-(acetyl) xylosyl]-xyloside	0.05±0.01	0.08±0.01
Cyanidin-3-O-xyloside	0.62±0.02	3.37±0.02
Cyanidin-3-gentiobioside	40.57±1.05	126.58±1.49
Cyanidin-3-O-arabinosidase-glucoside	0.18±0	0.76±0.04
Cyanidin-3-xylosyl-galactoside	0.15±0	0.71±0.05
Cyanidin-3-O-glucoside	525.33±23.44	1683.98±20.78
Cyanidin-3-malonyl-succinyl-succinyl-glucoside-glucoside	N/A	0.04±0
Cyanidin-3-O-(6"-O-acetyl-2"-O-xylosyl) glucoside	0.01±0	N/A
Cyanidin-3-O-glucoside-5-O-galactoside	0.01±0	0.03±0
Cyanidin-3-O-arabinoside	0.03±0	0.11±0.01
Cyanidin-3-O-(6-O-p-coumaroyl)-glucoside	0.59±0.03	0.32±0
Cyanidin-3,5-O-diglucoside	40.41±1.76	127.55±1.03
Cyanidin-3-O-(malonyl)(glucoside)galactoside	0.03±0.01	0.12±0.01
Cyanidin-3-dimalonyl-glucoside	0.57±0.04	N/A
Cyanidin-3-O-(6-O-malonyl-beta-D-glucoside)	1.63±0.12	N/A
Cyanidin-3-O-(6"-O-coumaryl-galloy) glucoside	0.04±0.01	0.03±0
Cyanidin-3-O-(hydroxybenzoyl)glucoside	0±0	0.01±0
Cyanidin-3-O-sophoroside	0.22±0.02	1.71±0.08
Cyanidin-3-[6"-(Galloy)rhamnoside]	N/A	0.03±0
Cyanidin-3-xylosyl-glucoside	0.13±0	0.59±0.04
Delphinidin-3-O-sophoroside	4.73±0.12	N/A
Delphinidin-3-O-(6"-O-caffeoyl) glucoside	N/A	0.02±0
Delphinidin-3,5-O-diglucoside	4.8±0.22	24.64±0.17
Delphinidin-3-O-(6-O-p-coumaroyl)-glucoside	N/A	0.02±0
Delphinidin-3-O-sambubioside	0.03±0.01	N/A
Delphinidin-3,5,7-Triglucoside	0.27±0.03	N/A
Delphinidin-3-O-galactoside	68.21±2.25	280.45±3.59
Delphinidin-3-O-glucoside	65.83±3.52	279.03±3.35
Delphinidin-3-O-(6-O-acetyl)-glucoside	0.19±0.03	0.42±0.02
Malvidin-3-O-(6"-O-feruloyl) xyloside	0.1±0.01	N/A
Malvidin-3-O-(6"-O-coumaroyl) xyloside	N/A	0.13±0
Malvidin-3-O-(6"-acetylglucoside)-5-glucoside	0.17±0.02	0.11±0
Malvidin-3-O-sambubioside	0.06±0.01	0.11±0.02
Malvidin	N/A	0.02±0
Malvidin-3-O-glucoside	385.48±30.53	1317.77±11.6
Malvidin-3-O-galactoside	408.01±35.41	1313.41±10.17
Malvidin-3,5-O-diglucoside	325.96±15.9	486.99±3.55
Malvidin-3-O-rhamnoside	N/A	0.54±0.01
Malvidin-3-O-arabinoside	0.07±0	0.26±0.01
Pelargonidin-3-O-rutinoside	0.94±0.01	1.77±0.17
Pelargonidin-3,5-O-diglucoside	3.54±0.2	7.98±0.21
Pelargonidin-3-O-coumaroyl-5-O-galactoside	0.96±0.07	1.96±0.1
Pelargonidin-3-O-(acetyl)(malonyl)glucoside	0.05±0	0.28±0
Pelargonidin-3-O-glucoside	10.51±1.12	70.16±0.58
Peonidin-3-O-sambubioside	0.36±0.03	0.49±0.02
Peonidin-3-O-arabinoside	0.03±0	0.09±0

Peonidin-3,5-O-diglucoside	652.28±30.79	936.09±10.01
Peonidin-3-O-(acetyl)(malonyl)galactoside	N/A	0.04±0
Peonidin-3,5-diglucoside	303.82±8.69	413.92±5.57
Peonidin-3-O-sophoroside	N/A	3.41±0.08
Peonidin-3-O-glucoside	1343.73±54.38	3338.72±53.77
Peonidin-3-O-(caffeoyl)rhamnoside	0.02±0	N/A
Peonidin-3-O-(6-O-p-coumaroyl)-galactoside	0.14±0.01	N/A
Peonidin-3-O-5-O-(6-O-coumaroyl)-diglucoside	0.2±0.02	N/A
Petunidin-3-O-galactoside	47.28±0.85	185.57±5.06
Petunidin-3-O-arabinoside	N/A	0.02±0
Petunidin-3-O-glucoside	131±7.42	342.31±2.75
Procyanidin C1	17.03±2.48	119.14±11.29
Procyanidin B3	114.03±2.47	72.79±2.36
Procyanidin B1	116.06±2.88	71.22±0.93
Procyanidin A2	0.17±0.03	0.61±0.05
Procyanidin B4	32.83±1.06	128.94±5.66
Procyanidin B2	64.4±1.88	271.84±3.45

Table S7 Glycosylation modification of anthocyanin components

anthocyanin components	WT (μg/g)	OE1 (μg/g)
3-O-glucoside	2461.8873	7031.9658
3-O-galactoside	523.5026	1779.4253
3,5-O-diglucoside	1026.9934	1583.2597
3,5-diglucoside	303.8229	413.9210
3-gentiobioside	40.5682	126.5787
3-O-sophoroside	4.9487	5.1265
3-O-xyloside	0.6197	3.3683
3-O-coumaroyl-5-O-galactoside	0.9564	1.9552
3-O-rutinoside	0.9353	1.7723
3-O-arabinosidase-glucoside	0.1782	0.7579
3-O-(6-O-malonyl-beta-D-glucoside)	1.6306	0.0000
3-xylosyl-galactoside	0.1529	0.7071
3-O-sambubioside	0.4511	0.5989
3-xylosyl-glucoside	0.1346	0.5868
3-O-rhamnoside	0.0000	0.5387
3-O-arabinoside	0.1317	0.4837
3-O-(6-O-acetyl)-glucoside	0.1855	0.4227
3-O-(6-O-p-coumaroyl)-glucoside	0.5917	0.3507
3-dimalonyl-glucoside	0.5726	0.0000
3-O-(acetyl)(malonyl)glucoside	0.0487	0.2773
3,5,7-Triglucoside	0.2661	0.0000
3-O-5-O-(6-O-coumaroyl)-diglucoside	0.2038	0.0000
3-O-(6"-acetylglucoside)-5-glucoside	0.1695	0.1112
3-O-(6-O-p-coumaroyl)-galactoside	0.1386	0.0000
3-O-(6"-O-coumaroyl) xyloside	0.0000	0.1336
3-O-(malonyl)(glucoside)galactoside	0.0257	0.1183
3-O-(6"-O-feruloyl) xyloside	0.0967	0.0000
3-[6"-(acetyl) xylosyl]-xyloside	0.0455	0.0793
3-malonyl-glucosyl-glucoside	0.0190	0.0609
3-O-(acetyl)(malonyl)galactoside	0.0000	0.0429
3-O-(6"-O-coumaryl-galloy) glucoside	0.0368	0.0252
3-[6"-(Galloy)rhamnoside]	0.0000	0.0336
3-malonyl-succinyl-succinyl-glucoside-glucoside	0.0000	0.0359
3-O-glucoside-5-O-galactoside	0.0108	0.0260
3-O-(caffeoyl)rhamnoside	0.0189	0.0000
3-O-(6"-O-caffeoyl) glucoside	0.0000	0.0156
3-O-(hydroxybenzoyl)glucoside	0.0039	0.0127
3-O-(6"-O-acetyl-2"-O-xylosyl) glucoside	0.0058	0.0000

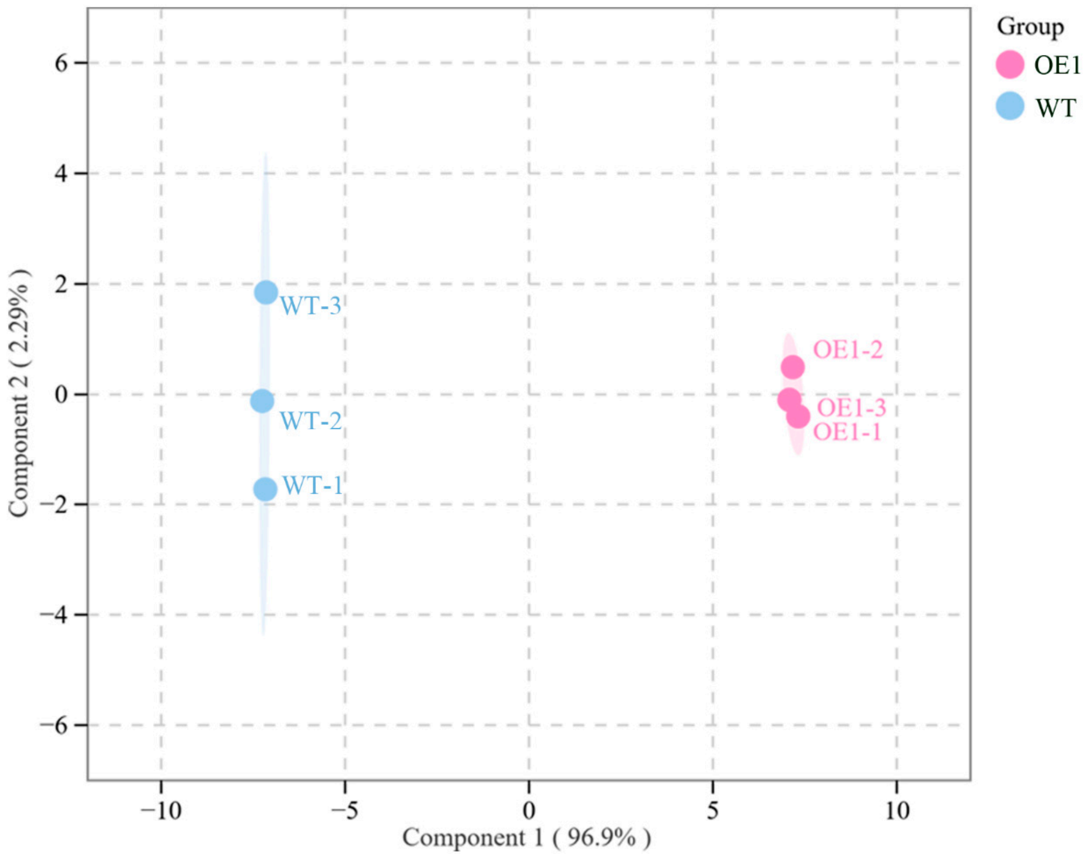

Figure S1 OPLS-DA score map of metabolites PCA of metabolome data. The x-axis represents principal component 1 (PC1); the y-axis represents principal component 2 (PC2)

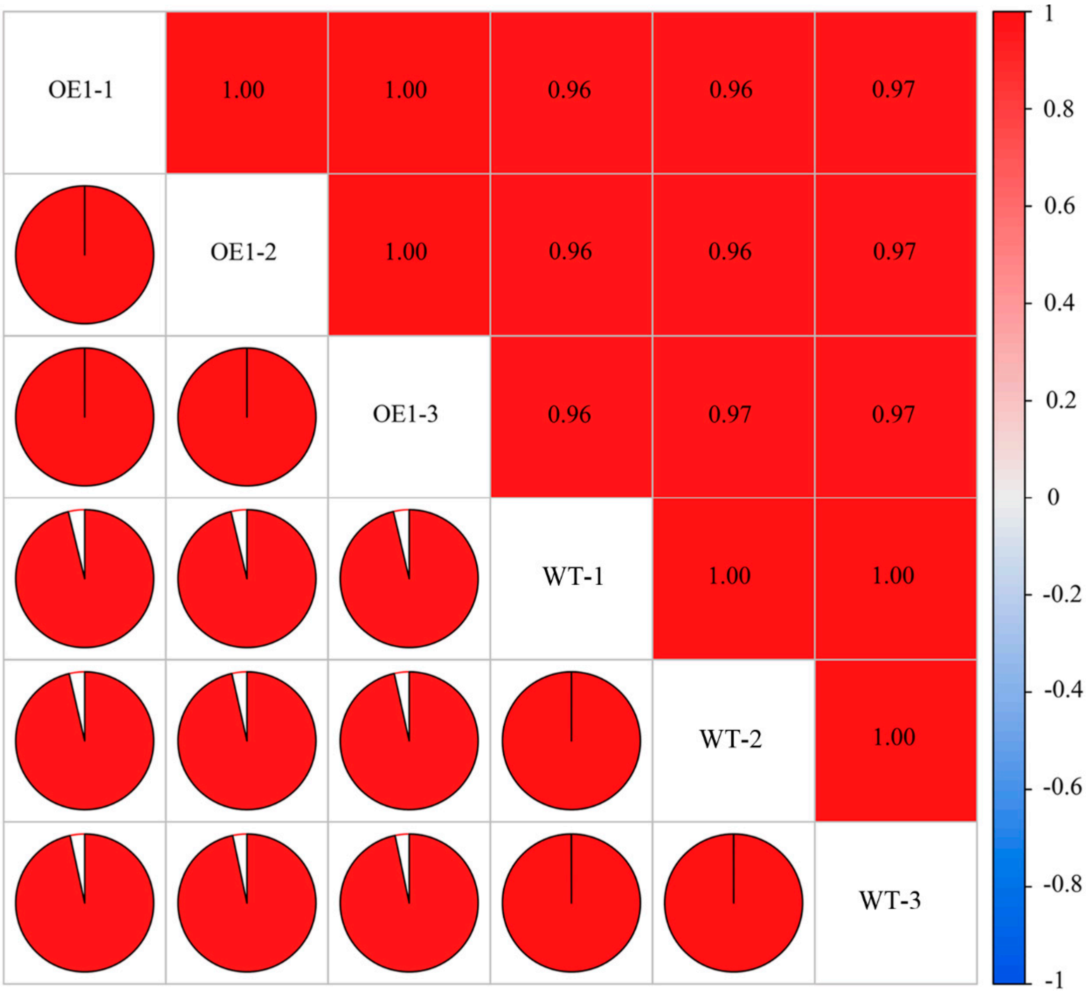

Figure S2 Pearson's correlation analysis of each sample in the transcriptome data

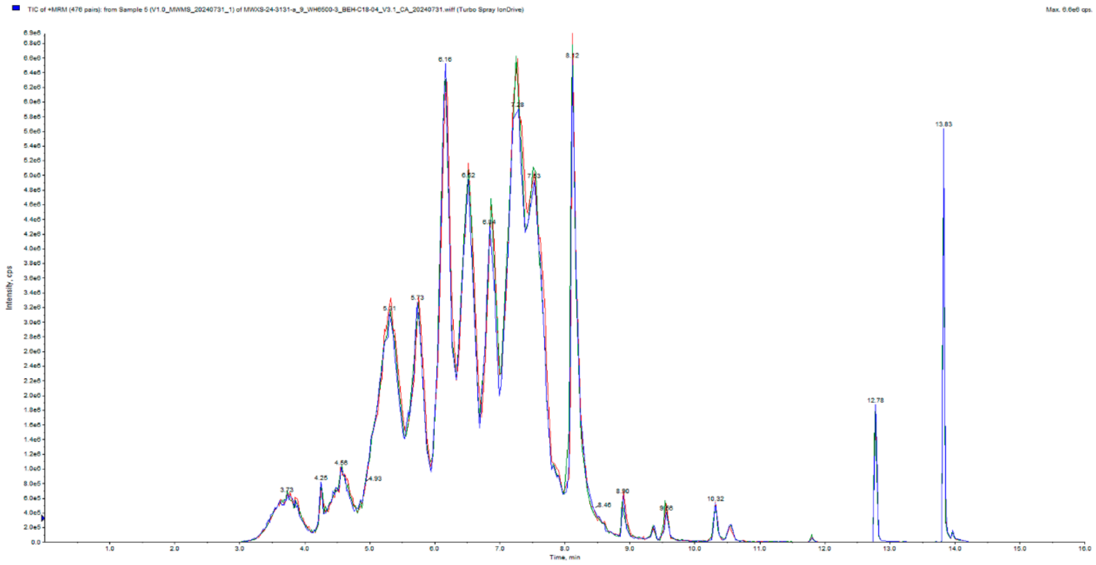

Figure S3 Superposed graph of total ions current in quality control samples
